# Retrofitting the BAC cloning vector pBeloBAC11 by the insertion of a mutant *loxP* site

**DOI:** 10.1186/s13104-017-2631-8

**Published:** 2017-07-28

**Authors:** Jonathon S. Coren

**Affiliations:** Biology Department, Elizabethtown College, One Alpha Drive, Elizabethtown, PA 17022 USA

**Keywords:** pBeloBAC11 cloning vector, Human genomic libraries, *loxP* sites, Transposon-mediated deletions

## Abstract

**Objective:**

Human genomic libraries constructed in bacterial artificial chromosome vectors were utilized to make physical maps of all 23-chromosome pairs and as the templates for DNA sequencing to aid in the completion of the Human Genome Project. The goal of this study was to modify the BAC vector pBeloBAC11 so that genomic inserts contained in this vector could be subjected to bidirectional transposon-mediated nested deletions from the wild-type and mutant *loxP* sites present.

**Results:**

An oligonucleotide containing a mutant *loxP* 2272 site and a *Xho*I restriction enzyme sequence was designed and inserted at the *Sfi*I restriction site located approximately 200 basepairs upstream of the *lacZ* gene in pBeloBAC11. Clones containing the desired insert were identified by *Xho*I restriction digests since an additional band was generated. This transposon-mediated deletion technology allows researchers to identify the boundaries of *cis*-acting elements and genes.

## Introduction

Bacterial artificial chromosome (BAC) and P1 artificial chromosome (PAC) vectors were used to generate human genomic libraries for the Human Genome Project (HGP) since they could accommodate inserts up to 300 kilobases (kb) and did not generate chimeric clones [1, 2]. Since the PAC vectors were derived from the plasmid pAd10sacBII, they all contained a *loxP* site that is part of the bacteriophage P1 genome [3, 4].

A mini-Tn10 transposon plasmid containing a *loxP* site was developed to retrofit P1 clones to generate nested deletions [5]. We then used this technology to demonstrate that large nested deletions could be generated in both PAC and BAC clones [6]. A study by Lee and Saito [7] investigated the role that each nucleotide in this 34-bp sequence plays in the recombination process. They identified two double-base substitutions in the 8-bp spacer region (5171 and 2272) that efficiently recombined with an identical mutant but not with the other mutant or the wild-type *loxP* site.

The goal of this study was to modify the BAC cloning vector pBeloBac11 to make it more versatile for a variety of studies. Oligonucleotides containing the 2272 version of the mutant *loxP* site and an internal *Xho*I restriction sequence were designed and ligated into the *Sfi*I site of this BAC vector. Researchers can transform transposon plasmids carrying either a wild type or mutant *loxP* site into the *E. coli* cells containing any BAC clone of interest created in pBeloBac11 to generate transposon-mediated nested deletions from both ends of the genomic DNA.

## Main text

### Methods

The following oligonucleotides were synthesized and gel purified (Sigma-Genosis): 5′CTCGAGATAACTTCGTATAAAGTATCCTATACGAAGTTATCCC3′ and 5′ATAAACTTCGTATAGGATACTTTATACGAAGTTATCTCGAGGGG3′. Each oligonucleotide was dissolved in 10 mM Tris–Cl, 1 mM EDTA, pH 8.0 (TE) at a final concentration of 1 μg/ml and incubated at 4 °C overnight. The oligonucleotides were combined in the presence of 50 mM NaCl and heated to 80 °C for 5 min and then slowly cooled to room temperature.

A 500-fold molar excess of the double-stranded oligonucleotide was incubated with pBeloBac11 DNA that had been digested with *Sfi*I for 2 h at 37 °C (New England BioLabs) in the presence of 1 U of T4 DNA ligase (Invitrogen) at 15 °C overnight. The ligation reaction was heat inactivated at 70 °C for 15 min and then spot dialyzed against 0.5X TE for 1 h. Two μl aliquots of the ligation reaction were mixed with 25 μl of electrocompetent DH10B cells (Invitrogen), placed into a 1 cm gap cuvette (BTX) and electroporated at 2.5 kV and 129 Ohms using the BTX Electro Cell Manipulator 600. The cells were allowed to recover in 1 ml SOC media (Invitrogen) at 37 °C and 225 rpm for 1 h and then 100 μl aliquots were spread onto LB + 34 μg/ml CM (Sigma) plates. Individual colonies were grown overnight in 2.5 ml of LB + CM media and DNA was generated using an alkaline lysis miniprep procedure [8]. The DNA samples were digested with 10 U of *Xho*I for 2 h at 37 °C (New England BioLabs) and electrophoresed in a 0.8% agarose gel in 0.5X TBE.

### Results

**Fig. 1:**
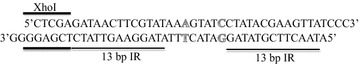
Design of the mutant *loxP* oligonucleotide. The 13 bp inverted repeats are *underlined*. The mutated bases in the 8 bp central spacer are shown in shadowed *grey font*. The *Xho*I site is also indicated

Since the BAC cloning vector pBeloBAc11 was commercially available from New England BioLabs, I decided to modify this plasmid to increase its utility for genomics studies. Two complementary oligonucleotides containing the 2272 version of the mutant *loxP* site were designed and synthesized. I also incorporated a *Xho*I restriction site at the 5′ end of the CTCGAGATAACTTCGTATAAAGTATCCTATACGAAGTTATCCC oligonucleotide and the 3′ end of the complimentary oligonucleotide to allow for identification of successful insertion of the mutant *loxP* site into pBeloBac11, which contains a single *Xho*I site (Fig. 1). Then the oligonucleotides were combined and heated to 80 °C and allowed to slowly cool to room temperature so that annealing would occur.

**Fig. 2:**
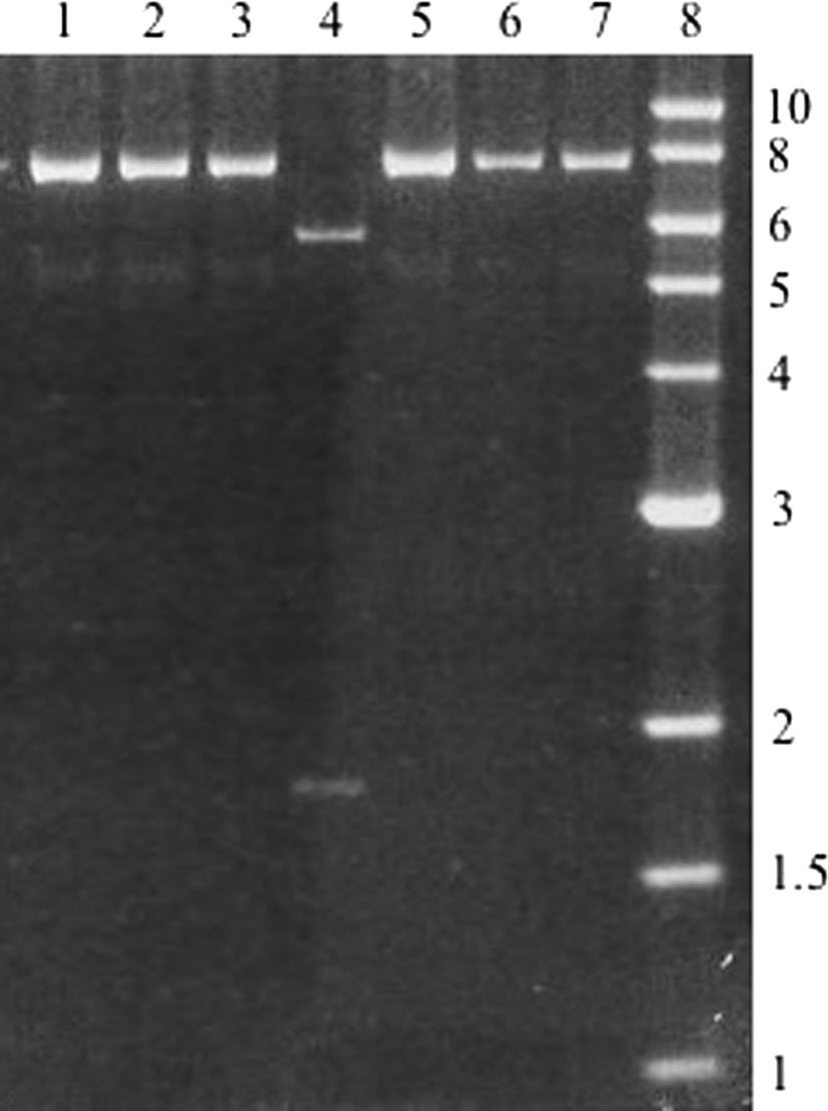
*Xho*I digests of miniprep DNA. Each DNA sample was digested with *Xho*I and electrophoresed on a 0.8% agarose gel. *Lanes 1–7* represent various transformants. *Lane 8* is a 1 kb ladder

**Fig. 3:**
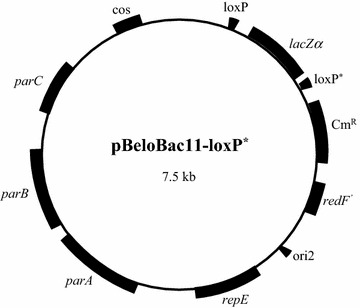
The pBeloBac11-loxP* vector. The mutant 2272 *loxP* site was inserted upstream of the *lacZ* gene

A 500-fold molar excess of the double stranded oligonucleotide was ligated to *Sfi*I-digested pBeloBac11 and the ligations were electroporated into DH10B cells and spread onto LB + chloramphenicol (CM) plates. I obtained miniprep DNA from several transformants, digested them with *Xho*I and analyzed the clones by agarose gel electrophoresis. The desired clone would generate two bands of 5.8 and 1.7 kb, while the religated vector would generate a single band of 7.5 kb. I identified several clones with the desired mutant *loxP* site; one of these transformants is shown (Fig. 2, lane 4). This newly created pBeloBac11-loxP* vector is illustrated (Fig. 3).

### Discussion

**Fig. 4:**
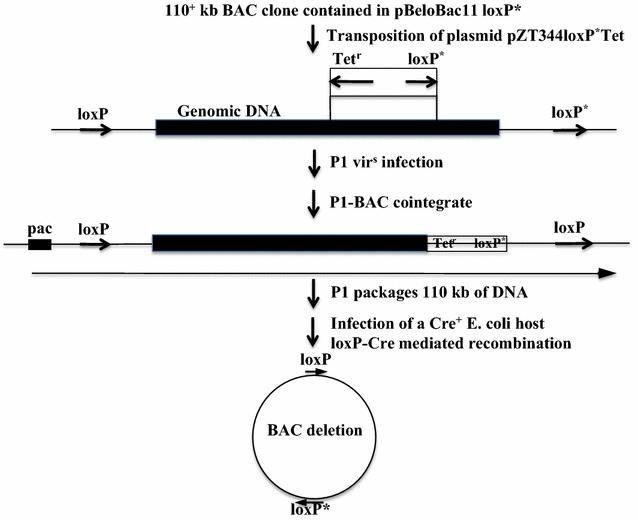
Scheme for recovery of BAC deletions. The transposon plasmid is introduced into the cell harboring the BAC clone of interest. Random transposition of the mini Tn10 cassette into the BAC clone is initiated upon the addition of IPTG. P1*vir* ^*s*^ infection results in the deletion of the DNA between two mutant *loxP* sites in the same orientation and formation of a P1-BAC cointegrate. P1 packaging initiates at the *pac* site until the capsid is full. Infection of the Cre^+^ strain NS3516 results in cyclization of the BAC clone

*Xho*I digests of the transformants resulting from the ligation of the mutant loxP oligonucleotide to pBeloBac11 demonstrated that the modified vector was generated. Nested deletions emanating from the mutant l*oxP* site of the BAC clones from the pilot genomic library that was constructed in this study can be generated using the mini-transposon system that we employed earlier (Fig. 3) [6]. First, the mini-transposon vector containing the mutant *loxP* site must be transformed into the BAC clone of interest. Upon addition of IPTG, the mini-Tn10 transposon cassette will randomly integrate into the BAC clone. When these cells are infected with a lytic strain of the bacteriophage P1, then deletions will occur between the two mutant *loxP* sites as long as they are in the same orientation (Fig. 4) [5]. Alternatively, existing BAC clones can be digested with *Not*I to liberate the genomic DNA. Then the desired fragment could be ligated into *Not*I-digested pBeloBac11-loxP^*^. Deletions emanating from the wild-type l*oxP* site can be generated using the transposon plasmid pTnPGKpuro/loxP-EBV; this would also modify the BAC clone so that it also can be propagated in mammalian cells since it would contain the Epstein Barr Virus (EBV) latent origin of replication *oriP* and the trans-activating gene EBNA-1 [9]. Utilization of this technology will allow researchers to identify both boundaries of *cis*-acting elements and genes.

### Limitations

- pBeloBAC11 loxP^*^ cannot be propagated in mammalian cells.
